# Accessing and engaging with antenatal care: an interview study of teenage women

**DOI:** 10.1186/s12884-021-04137-1

**Published:** 2021-10-10

**Authors:** Anna Wong Shee, Natasha Frawley, Carolyn Robertson, AnneMarie McKenzie, Julie Lodge, Vincent Versace, Cate Nagle

**Affiliations:** 1 Primary and Community Care, Ballarat Health Services, Ballarat, Victoria 3350 Australia; 2 Deakin Rural Health, Deakin University, Warrnambool, Australia; 3 James Cook University, Centre for Nursing and Midwifery Research, 1 James Cook Drive, Townsville, 4814 Australia; 4 Townsville Hospital and Health Service, 100 Angus Smith Drive, Douglas, QLD 4814 Australia

**Keywords:** Adolescent pregnancy, Rural health, Prenatal care, Maternal health services

## Abstract

**Background:**

Pregnant teenagers in rural and regional areas experience distinct disadvantages, that are not simply a function of their age, and these have a substantial impact on their health and that of their baby. Studies demonstrate that antenatal care improves pregnancy outcomes amongst pregnant women, especially adolescents. Understanding teenager’s views and experiences of pregnancy and motherhood is important to ensure antenatal care meets young women’s needs. This study explored teenage women’s experiences and perceptions of barriers and facilitators to engaging in pregnancy care in rural and regional Victoria, Australia.

**Methods:**

Between February–October 2017, pregnant women aged ≤19 years were purposively recruited from one regional and two rural health services in Victoria. Semi-structured, face-to-face interviews guided by naturalistic inquiry were conducted and an inductive approach to analysis was applied.

**Results:**

Four key themes emerged from the analysis of the transcripts of 16 interviews: Valuing pregnancy care, Interactions with Maternity Service, Woman-centred care, and Support systems. Teenage women primary motivation to attend care was to ensure their baby’s wellbeing and lack of engagement occurred when the relevance of antenatal care was not understood. Appointment flexibility and an accessible location was important; most participants were reliant on others for transport. Continuity of carer and respectful, non-judgement communication by staff was highly valued. Many young women had fractured families with pregnancy diminishing their social world, yet having a baby gave them purpose in their lives.

**Conclusion:**

Maternity services and health professionals that provide flexible, adaptable women-centred care and support through pregnancy and early motherhood will assist young women’s engagement in antenatal care.

**Supplementary Information:**

The online version contains supplementary material available at 10.1186/s12884-021-04137-1.

## Background

Pregnant teenagers are often vulnerable, face unique challenges in seeking health care and their pregnancies are at increased risk of complications and poor outcomes [1, 9]. Women aged 15–19 years have twice the risk of dying from pregnancy-related causes and a 50% higher risk of stillbirth compared to women aged 20–29 years [1, 29]. There are several factors that increase the risk of poor outcomes for pregnant teenagers: low socio-economic status and social stigma [4, 19]; lack of social support [11]; poor antenatal attendance [1, 18]; smoking and substance abuse [14, 18]; and biological factors including inadequate maternal weight gain [17]; and biological immaturity [24]. Pregnant teenagers experience distinct disadvantages and these have a substantial impact on their health and that of their baby [1, 18].

Studies demonstrate that antenatal care improves pregnancy outcomes amongst all pregnant women, especially adolescents [28, 29], and evidence supports the importance of care early in pregnancy [29]. The Australian Government’s “Clinical Practice Guidelines: Pregnancy Care” [7] recommend that the first antenatal visit occur within the first 10 weeks of pregnancy. Commencing regular antenatal care in the first trimester is associated with better maternal health in pregnancy, fewer interventions in late pregnancy and positive child health outcomes [1, 2].

Pregnant teenagers often have inadequate antenatal care as they tend to register for care at a later gestation and attend fewer appointments than women aged 20–24, or receive no antenatal care [1]. Delays in seeking care for complications during pregnancy, increase the risk of maternal and foetal morbidity and mortality [22]. Research has shown that young women’s perceptions and expectations of service providers underlie many of the barriers to accessing care [16]. Models of antenatal care need to recognise and be responsive to the disparate needs of pregnant teenagers [18, 29].

Overall, births to teenage women have been declining, however they are over-represented in in rural communities and socially disadvantaged areas [11]. The latest Organization for Economic Co-operation and Development (OECD) report, based on 2015 data, shows that while Australia’s birth rate for 15–19 year old women was similar to the OECD average [21], pregnant teenage women were more likely to live in regional areas (13 births per 1000 women aged 15–19) than in metropolitan regions (7 births per 1000) [6]. Birth rates for teenage women are not consistent across the Australian population and increase with geographical remoteness [1]. Pregnant teenagers living in rural and regional areas experience greater health inequities [1] compared with their urban counterparts and face unique challenges in accessing and engaging in antenatal care.

Understanding young women’s views and experiences of pregnancy and motherhood is important to ensure interventions to improve access and engagement with antenatal care meet young women’s needs [8, 29]. However, the literature on young women’s experiences of maternity care is limited, particularly for those living in rural and regional areas. The aim of our study was to explore teenage women’s experiences of engaging in pregnancy care in rural and regional Victoria, Australia.

## Method

This qualitative study was conducted at one regional and two rural health services in western Victoria, Australia. This study involved semi-structured, face-to-face interviews guided by naturalistic inquiry [15], applying an inductive approach to analysis.

### Participants

Purposive sampling of pregnant women 13–19 years of age attending antenatal clinics at the participating health services between February and October 2017 was utilised. Information about the project was provided to women within the age range when they attended the antenatal clinics. Partners and supports persons were included in the interview if requested by the participant. Women were screened by a midwife and excluded if there were concerns about the viability of their pregnancy or the complexity of the case.

### Data collection

Individual semi-structured interviews (Attachment 1) were employed and questions were pre-tested with three women and modified based on feedback prior to study commencement. Interviews were audiotaped and professionally transcribed, field notes were also taken. All the interviews were conducted by an experienced investigator (CN) who was not involved in clinical care. The interviewer (CN) is a female registered nurse and midwife with a PhD, who holds a co-joint university and health service appointment and whose research interests include women’s experience of maternity care.

The interviews were conducted at a mutually convenient locations which included the clinic and participants’ homes.

### Data analysis

Transcripts were analysed by two researchers (AWS, CN) independently using thematic analysis guided by Braun and Clarke’s approach [5]. The investigator (AWS) assisting with analysis is a female registered physiotherapist, who holds a co-joint university and health service appointment and whose research interests include health services research and research translation. An inductive approach was used with the coded categories derived directly from the transcript data [12]. Coding proceeded iteratively and related comments were grouped into themes. Approaching saturation of themes, interpretations of the data were discussed between investigators. Adaptations to themes, until stable themes were agreed, were made by consensus and involvement of a third coder was not required. Transcripts were not returned for member-checking.

Trustworthiness of the findings was enhanced through the following means: each interview was transcribed verbatim allowing the reader to determine the appropriateness of the interpretation provided by the researcher; and the analytical procedure of interpretative descriptive coding of each transcript through regular consultation between investigators, ensured that the findings were justified by the data.

### Ethical considerations

Women aged less than 18 years of age were deemed competent to consent if they meet the Gillick competency and the Fraser guidelines [20]. Prior to each interview, written consent was obtained. Ethics approval for the study was obtained from Ballarat Health Services and St John of God Human Research Ethics Committee.

## Results

Sixteen women were purposively selected based on the selection criteria. All of the women approached agreed to participate and there were no drop outs. All participants were English speaking, Caucasian and aged between 16 and 19 years. Twelve participants lived in a regional centre and four participants in rural towns. Two participants were attending school. Four of the participants were having their second child, two of which had previous pregnancy related complications. Four participants had their partners participate in the interview, one included her mother. The interviews were on average 14.5 min, with a range of 9.4 min to 22.5 min.

Four themes (Table 1), emerged: perceived value of pregnancy care; interactions with the maternity service; provision of woman-centred care; and their support systems during their pregnancy.

**Table 1:** Factors influencing pregnant teenagers’ engagement with antenatal care

Themes	Definition	Sub themes
Valuing pregnancy care	Motivation to attend driven by the value that they place on antenatal care	• Fetal and/or maternal wellbeing • Wanting to learn (valued the education given)
Interactions with Maternity Service	Experience with access to the maternity service and the antenatal clinic environment	• Environment – physical and culture • Access • Flexibility of appointments
Woman-centred care	The individualization of care and the extent to which they felt they were valued as a person	• Participation in care • Communication and IP skills of clinicians • Continuity of Carer • Information preference
Support systems	Life outside of the maternity service and the challenges of a lack of support	• Family and friends • Social isolation/challenges

### Valuing pregnancy care

The participants all recognised the importance of antenatal care and identified concern for the wellbeing and health of their baby as a primary motivation for attending antenatal care. Many of the women emphasised the need to ensure that their baby’s interests were served above their own, as highlighted by one woman’s comment.

I don't really say no to it because it's more so not for me (sic) it's for the baby's health. Yeah, I don't really have a right to say no (Participant 8, regional).

While all of the women were motivated to attend antenatal care, some of the women reported only recognising the value of antenatal care following complications in a previous pregnancy. One of the women talked about the lack of awareness and motivation to attend antenatal care during her first pregnancy in which her baby died.

… But with losing my first and then the becoming pregnant so quickly after that one, I was like well I need to knuckle down and go more often. (Participant 4, regional)

These women talked about a lack of understanding of the relevance of antenatal care to their age group and a perception that teenagers are not susceptible to pregnancy-related complications.

Participants commented that teenage women may be reluctant to attend antenatal clinics because they perceived care providers as being authoritarian and they disliked being told what to do. The women also suggested that teenage women may not engage with antenatal care due to fear or denial. Some of the women commented that at times they had feeling self-conscious and uncomfortable if they perceived people were staring at them or singling them out. Feelings of shame and stigma associated with pregnancy can delay teenagers seeking antenatal care and getting important screening and education early in pregnancy.

… sometimes you feel like people are going to judge you because of how young you are, and because you’re not married, you’re not with your baby daddy (sic) or whatever they’re called. So, you have that resentment of coming to somewhere where people are going to stare at you be like what the hell. Why are you here so young? (Participant 12, rural)

Several of the women commented on the need to filter the amount and nature of information to avoid overwhelming themselves and increasing their anxiety. Some of the women avoided childbirth education because they found it scary and would rather cope with events as they arise. These women suggested that providing chunks of information relevant to their stage may make it easier for women to digest and cope.

I try not to - but I try to get a little bit of information, but not too much; not enough to like freak myself out, and not enough for me to be like oh - and like, yeah, it's just doing my head in and stuff. (Participant 7, regional)

Several of the participants identified the influence of their family and friends on their decision to participate. One woman’s partner described how family support could influence antenatal attendance.

“Also like I'm speaking on the partner's behalf, sometimes a partner might decide that they're too tired from work and that sort of thing because there's been a couple of appointments where I haven't been able to make it to … I feel as though some partners might say you're fine, there's no need to go, the baby's kicking. (Partner- Participant 9, regional)

Women reported finding their antenatal appointments interesting and they valued the ability to follow their baby’s milestones. Several women commented that hearing their baby’s heartbeat motivated them to attend as they found it both reassuring and enjoyable. Some of the women identified the need to monitor their own health during pregnancy. They also valued the antenatal support in managing their pregnancy and in preparing for parenthood.

### Interactions with maternity service

The young women reported valuing the respectful, welcoming demeanour of maternity service staff and trusting them to have the best interests of both their baby and the women at heart.

Just the friendly staff, they've always got a smile on their face. Yeah, it's always warming to come in here. They just - they've just got that friendly sort of vibe about them so I know that I can trust them. (Participant 11, regional)

The young women were positive about the physical environment in the maternity services, appreciating the layout that catered for children and the calm environment. One of the women suggested a simple change to the antenatal clinic waiting room would make it easier for women to engage with each other.

I would just love for … the mums to be more involved. I know not everyone does want to be involved but maybe the chairs, they all sit facing one way. If they were sitting facing a different way you might be more likely to start a conversation with the lady sitting over there. (Participant 9, regional)

The women reported a variety of geographical, financial, social, and scheduling barriers and facilitators to accessing antenatal care. None of the women had driving licenses and relied on family, friends or public transport to get to appointments.

She said that it costs nothing, and we were right to get it, and she bulk billed us. Then we got the bill for the company that she used. If I knew that it was going to charge us, even if it was going to charge us a little bit, I would have, that would have been fine. But $135, we just couldn't afford it at the time.” (Participant 3, regional)

The women identified choices for appointment times and the option to manage appointments via text and fit in with their school schedule as facilitators to attending antenatal care.

I usually get an appointment in the morning so then I'm out of here by the time, lunch time at school, so I can get back to school but I think they're pretty good. (Participant 1, rural)

### Woman-centred care

Most of the women had a sense of individualized care and felt listened to and comfortable asking questions. The young women valued being given choices and being included in the decision-making around their care.

I'm always let known of the things that they're going to do and the decisions they're going to make, and they're always like is there anything that you want to input? Yeah, I definitely feel very included in what goes on with the care here. (Participant 4, regional)

Health professionals’ ability to communicate in a non-judgmental and non-threatening manner was important to the women. One young woman compared her experience with her current midwife, who was dedicated to working with young mums, to her previous antenatal care. Her comments highlighted that the delivery and content of information is key to engaging with young women during their pregnancy.

I don't know it's just the vibe and she's [Young Mum’s midwife] always got a smile and is always polite. She doesn't come across creepy or anything. About two and a half years ago when I had my first one I had this old lady that was my midwife for like two days. She brought out these - it was like a little cushion diagram of the vagina and how the baby comes out and she scared the shit out of me. It's just a bit more comfortable with [Young Mum’s midwife] than the other one. (Participant 8, regional)

The midwives’ communication skills contributed to the young women’s perceptions of an equal power relationship. The women appreciated not being pressured in to making decisions and the ability of staff to value their questions. Many women found the use of texting invaluable, allowing them to get timely responses and reassurance when they needed it.

… They don't sit … and act like they've got more knowledge. I mean they clearly do, but they sit there and communicate with you and they speak in way that you're able to understand instead of just reading what they would have read from a textbook to you. (Participant 9, regional)

All of the young women valued knowing the midwives and doctors and that the care was consistent.

### Support systems

Many of the young women came from fractured families and had experienced social isolation that impacted on their confidence to attend childbirth education and maternal and child health centres as they felt uncomfortable in group situations. A desire for connections with other young mothers was common.

… after I had my son and you go to see the maternal child health nurse and they try and get you to go to a mothers’ group, I chose not to because I was scared about going there and being the youngest person there, and everyone being 30 and having a nice car and owning a house, and I was the odd one out. So, I didn't go. I wished that I would have had some more support system I guess … (Participant 13, rural)

Some of the women and their partners commented on their diminished social life and reliance on each other. One of the partners talked about his isolation from his family due to cultural and generational differences. He highlighted the value talking to another young partners for support.

It'd be good … for another young guy like me to be able to talk to them and say look how have you been able to cope? Have you found it stressful, have you adjusted yet? Just being able to talk like that. (Partner- Participant 9, regional)

All the young women felt that meeting with other young mothers would be beneficial to help them engage with antenatal care.

All participants had some form of support from family, friends, health professionals, support or support groups. Schools provided another form of assistance, providing information and enabling the young women to continue with their education.

… the school has been amazing. I couldn't thank them enough for the support they've given me. They're pretty much willing to do anything they can to help me finish school. (Participant 1, regional)

## Discussion

Participants identified challenges and motivations for attending antenatal care that related to valuing pregnancy care, interactions with the maternity service, women-centred care, and support systems.

Similar to a United States study investigating the motivators and barriers to antenatal care in pregnant teenagers, our study also found ‘concern for the health of their baby’( [27]) a strong motivating factors for antenatal clinic attendance. The women in our study, also identified additional benefits of antenatal care, such as enhanced knowledge, reassurance, validation and social support. A study by Michels (2000) found young women were motivated to engage in antenatal care because they valued the support in managing their pregnancy.

Some of the young women interviewed had not attended antenatal care for a previous pregnancy and had experienced adverse outcomes. They reported that their lack of engagement with antenatal care was due to several factors, including fear, denial, and a perception that antenatal care was not relevant for their age group. A French study exploring the relationship between anxiety and coping strategies during pregnancy found that anxiety in women during pregnancy was associated with inappropriate coping strategies, such as denial of reality and disengagement [10]. These findings may explain some women’s ambivalence towards antenatal care and highlight the potential to engage teenage women in care during pregnancy by recognizing and allaying issues of fear and denial.

Only a small number of teenage women in this study considered the value of antenatal care in terms of their own health. Pregnant teenagers face a number of health and social risks, such as anaemia, pre-eclampsia [31], stigma and violence by partners, parents and peers [32]. Little is known about the value teenage women place on antenatal care in terms of their own health. Further research is required in this area and there may be an opportunity to promote the value of antenatal care for maternal benefit.

All of the young women in this study were from rural and regional areas and faced challenges accessing antenatal care. Many of the young women found the use of texting invaluable, allowing them to schedule or make changes to appointments and receive timely responses and reassurance. The flexibility of appointments was important as none of the young women had driving licenses and relied on family, friends or public transport. Pregnant teenagers are more likely to birth in a public facility and to live in less economically advantaged areas than older women [23]. Enabling pregnant teenagers to obtain care or information quickly and easily, for example through texting, may improve engagement and provide a mechanism for opportunistic education.

The young women valued individualised care, being offered choices, and being included in the decision-making around their care. Women-centred care takes into account the women’s context and social determinants of health, and partners with women in their care [3]. A study on women’s perspective of antenatal care in Canada reported similar findings, with women preferring care providers who readily offered information, treated them as equals, and took the time to explain options thoroughly [26]. Feeling valued is a key to young women’s connection and engagement in antenatal care during pregnancy and critical in building their trust in health professionals.

The pregnant teenagers viewed one-to-one contact and follow-up by the same midwife as beneficial. The women that attended a dedicated young mum’s clinic valued the individualised care and the health professionals’ non-judgmental and non-threatening communication. The young women were grateful for staff that took the time to get to know them and they were able to discern when the care was not genuine. Continuity of carer in the maternity care has been strongly recommended and encouraged in Australia and worldwide [30]. The task that exists in rural and regional environments is providing a women-centred model of care, balanced with providing an accessible, sustainable service.

The young women and their partners spoke of diminishing social networks during pregnancy and limited support from family and friends. These findings are consistent with a Canadian study that found that teenage women had significantly lower social support than was optimal and lower than enjoyed by older women [13]. Social support, including emotional, social, and tangible help, has been identified as an important protective factor against stress for women in the maternal role [13]. The teenage women were positive about the support they received from their schools to continue with their education. The support of schools for pregnant teenagers is critical in enabling their continued school progress and long-term educational and career success [25]. Healthcare providers are encouraged to assess pregnant adolescents for feelings of social isolation and support their health and well-being during pregnancy, birth and in early parenthood.

The limitations of the current study include acknowledgement that the experiences of the young women included were those of pregnant teenagers who attended antenatal care. However, some of these women had not engaged in antenatal care with previous pregnancies and were able to provide retrospective accounts of their experiences of antenatal care and the barriers they faced. None of the participants in this project were from remote or very remote areas and the geographical remoteness in Victoria is not comparable to those in other Australian states and territories, thus these findings may not be generalisable to teenage women in other rural and remote areas. Additionally, only English speaking, Caucasian women were interviewed and their experience is likely to be different to other population groups e.g., indigenous teenage mothers (46.4 births per 1000) are substantially higher than non-indigenous teenage mothers (7.1 per 1000) [2].

Overall, the teenage women in this study were engaged with their antenatal care and positive about their maternity services. This is an important finding as it reflects a trust in the maternity services and healthcare professionals and support for self-efficacy in making decisions about pregnancy care.

## Conclusion

Maternity services and health professionals that provide women-centred care, support through pregnancy and afterwards, are flexible and adaptable to the unique needs of teenage women, encourage antenatal attendance for both the baby and the mother’s health, will all assist young women engage in antenatal care. These findings have informed the development of best practice guideline for teenage pregnancy care in a regional service. Maternity health professionals play a crucial role in providing pregnant adolescents with positive and encouraging perspectives of pregnancy and motherhood.

## Supplementary Information

**Additional file 1.**

## Data Availability

The datasets used and analysed during this study are available from the corresponding author on reasonable request. Access to anonymised data may be granted following review of the request. Exclusive use will be retained until the publication of major outputs.

## Abbreviations

OECD: Organization for Economic Co-operation and Development
