# Supplementary material for: Accessing and engaging with antenatal care: an interview study of teenage women
Source: BMC Pregnancy Childbirth. 2021 Oct 10;21:693. doi: 10.1186/s12884-021-04137-1 (PMC8504060; doi:10.1186/s12884-021-04137-1)
Supplement: Supplementary file 1 — Additional file 1. [file 12884_2021_4137_MOESM1_ESM.docx]

Attachment 1. Interview Questions

Interviewing teenagers on their antenatal care – determining barriers and facilitators to accessing care

Semi-structured interviews, one-to-one, not more than 30 minutes duration.

Introduction

Explain the purpose of the interview, confidential nature, and that the participant can cease the interview at any time.

Question 1: Warm up question

What aspects have you enjoyed most about your pregnancy care at the hospital?

Prompt – are there any aspects that they don’t enjoy?

Question 2

What motivates you to come to your appointments and scans?

Prompt – preference of timing/ location of appointments

Prompt – do they use the facebook page or SMS reminder system?

Question 3

What are the difficulties you have in accessing antenatal care?

Prompt – financial/ transport issues

Question 4

How would you describe the way you are treated during your pregnancy during your pregnancy care?

Prompt – are they able to participate in their care? Are they treated with respect? Do they feel their concerns are listened to? Are they able to ask questions?

Question 5

How confident are you in your healthcare providers and the hospital system?

Prompt – are there aspects you would like included in your care that are not currently?

Question 6

Who are your main supports?

Prompt – explore their role facilitating/ discouraging care

Question 7

Any other thoughts on how we can improve your care during pregnancy?
